# A mini-review on safeguarding global health amidst a “Pandemic” of armed conflicts

**DOI:** 10.1097/MD.0000000000037897

**Published:** 2024-05-17

**Authors:** Emmanuel Kokori, Gbolahan Olatunji, Ismaila Ajayi Yusuf, Timilehin Isarinade, Abdulrahmon Moradeyo Akanmu, Doyin Olatunji, Olumide Akinmoju, Nicholas Aderinto

**Affiliations:** a Department of Medicine and Surgery, University of Ilorin, Ilorin, Nigeria; b Department of Medicine and Surgery, Obafemi Awolowo University Teaching Hospital, Ife, Nigeria; c Department of Medicine, Ladoke Akintola University of Technology, Ogbomoso, Nigeria; d Department of Health Sciences, Western Illinois University, Macomb, IL; e Department of Medicine and Surgery, University of Ibadan, Ibadan, Nigeria.

**Keywords:** armed conflicts, global health, pandemic, public health

## Abstract

The year 2022 witnessed an alarming surge in state-based armed conflicts globally, reaching a staggering 56, with major hostilities in Ukraine, Myanmar, and Nigeria resulting in over 10,000 estimated conflict-related deaths. This trend continued with the onset of a significant conflict between Israel and Hamas in October 2023. The escalating frequency of armed conflicts, reaching the highest number since 1946, poses a critical threat to global health. This paper explores the multifaceted health impacts of armed conflicts, encompassing physical injuries, infectious diseases, malnutrition, and profound mental health consequences. Healthcare systems in conflict zones face severe strain, and achieving Sustainable Development Goals by 2030 becomes increasingly challenging. The surge in armed conflicts globally is characterized as a “pandemic,” justifying urgent attention. The paper identifies and discusses strategies to safeguard public health in conflict zones, emphasizing humanitarian response, protecting healthcare workers and infrastructure, building preparedness and resilience, and promoting mental health support. In navigating this “pandemic” of armed conflicts, comprehensive strategies are imperative to address the intricate challenges and secure a healthier global future.

## 1. Introduction

The year 2022 witnessed an alarming escalation in the number of active state-based armed conflicts, reaching a staggering 56, with major hostilities erupting in Ukraine, Myanmar, and Nigeria, each accounting for over 10,000 estimated conflict-related deaths.^[1]^ Adding to the global unrest, October 2023 marked the onset of a significant conflict between Israel and Hamas.^[2]^ Over the years, the incidence of such conflicts has shown an upward trend, with yearly recorded armed conflicts increasing from 17 in 1946 to 54 in 2016 and again in 2019. Notably, the figure surged to 56 in 2022, marking the highest number of yearly active armed conflicts since 1946.^[3,4]^ This unprecedented rise in armed conflicts spans across the Americas, Europe, Africa, and Asia, with Africa experiencing a higher prevalence.^[5]^ The Americas, on the other hand, recorded the least number of armed conflicts.^[6]^

Conflict intensification in regions like the Middle East has led to a staggering spike in war-related mortality, severely straining hospital capacities.^[5]^ Combatant and civilian casualties take precedence over regular patients, disrupting routine healthcare services. Countries such as Syria, Yemen, Iraq, South Sudan, and others have faced deliberate targeting of health facilities in recent years.^[5]^ Regardless of their nature – whether interstate, intrastate, or extra systemic – armed conflicts share common characteristics, involving organized groups engaged in intense armed fighting.^[7]^ The implications of armed conflicts on public and global health are profound, impacting areas such as trauma, infectious diseases, non-communicable diseases, sexual and reproductive health, child health, mental health, and nutrition.^[8]^ The consequences extend to violence, population displacement, infrastructure damage, disrupted public health services, food insecurity, and conflict-related deaths.^[9]^ Displaced populations, whether internal or as refugees, face heightened risks, with internally displaced persons being particularly susceptible to malnutrition and disease.^[8]^ In 2017, approximately 630 million women and children, constituting 10% of women and 16% of children globally,^[10]^ either experienced displacement due to conflict or lived in dangerously proximity to conflict events.

The impacts of armed conflicts on healthcare systems are profound and wide-ranging. Hospitals in conflict zones are strained, and routine healthcare services are disrupted as the urgency to treat combatants and casualties takes precedence.^[7]^ The deliberate targeting of health facilities exacerbates the challenges, resulting in a dire situation for civilians in need of medical attention. Furthermore, conflict-affected countries encounter challenges in achieving the Sustainable Development Goals (SDGs) by 2030, a critical milestone for global health.^[11]^ The recent surge in armed conflicts worldwide, with its grievous implications for public health, justifies characterizing this situation as a “pandemic.” This paper aims to identify strategies and opportunities to safeguard global health amidst what can be likened to a “pandemic” of armed conflicts.

## 2. Health impacts of armed conflicts

### 2.1. Physical health consequences

One of the direct consequences of armed conflict is physical injury, arising from the direct or indirect effects of instruments used in armed conflicts.^[12]^ Given the typically violent nature of armed conflicts, both combatants and noncombatants become casualties of war. Globally, data indicates that in 2013, over 800,000 individuals suffered injuries requiring hospitalization during conflicts, and nearly half of this number lost their lives to collective violence.^[13,14]^ Despite a gradual decline in this number over subsequent years, there was a significant and historic increase in conflict-related deaths in 2022, marking a rise of over 95%, with a total of 238,000 people losing their lives in global conflicts.^[15]^ During the Libya conflict, more than 16,000 reported deaths and over 42,000 injuries occurred over five years.^[16]^ These fatalities and injuries resulted directly from blasts and gunfire, causing harm that led to deaths and disabilities.^[17]^ The mortality rate is influenced by the intent of violence, such as genocide, and the types of weapons used, with actions like bombing and shelling causing more widespread destruction and mortality compared to one-on-one combat.^[18]^

War serves as a conduit for infections, especially during armed conflicts when people are compelled to relocate from one area to another, heightening their exposure to disease vectors.^[19]^ Displacement leads to overcrowding in new settlements, escalating the susceptibility to diseases such as diarrheal illnesses, respiratory infections, cholera, diphtheria, measles, and tuberculosis.^[20]^ These infectious diseases, considered communicable, emerge indirectly from conflict and are accountable for a significant portion of conflict-related deaths.^[21]^ Furthermore, the lack of access to clean food, water, shelter, and hygiene exacerbates the emergence of these diseases.^[22]^ Apart from the threats and disruptions to healthcare in these regions, conflicts also exacerbate already existing public health problems in the affected regions. Yemen is currently undergoing a humanitarian crisis that has left those with non-communicable diseases without access to proper healthcare, hence the massive mortality rates around the country.^[23]^ Some of the prevalent public health diseases in these regions record high rates of illness and mortality, with over 70% of cases of epidemic-prone illnesses like cholera, measles, and meningitis, 60% of maternal deaths that could have been prevented, 53% of deaths in children under the age of 5, and 45% of infant deaths.^[24]^

Children exposed to conflict experience an elevated vulnerability to malnutrition, primarily attributed to key socio-demographic factors. These factors explaining susceptibility to malnutrition include limited access to food, disruption of socioeconomic activities during armed conflict resulting in the loss of assets and an immediate threat to food security, leading to food inaccessibility.^[23]^ The increased infection rate during conflict exposes children, who are already more susceptible to illnesses, to acute illnesses, thereby contributing significantly to malnutrition.^[24]^ In conflict-affected areas, breadwinners face challenges in maintaining job commitments due to safety concerns, impacting families’ ability to afford food, which can be a crucial factor contributing to malnutrition.^[25]^

### 2.2. Mental health consequences

During armed conflicts, psychological trauma is prevalent and manifests through changes in mood, anxiety, and depression.^[26]^ Feelings of sadness and hopelessness are psychological effects that result from the impact of conflicts. This trauma arises from the direct effects of conflict, such as physical trauma, injury, loss of loved ones, loss of assets, and economic hardships.^[27]^ Another impact stems from the increased incidence of sexual violence during conflicts, with girls particularly vulnerable to exploitation, resulting in psychological trauma for them.^[28]^ Various forms of psychological trauma also arise from forced migration, interrupted education, neglect, imprisonment, exposure to infectious diseases, internal displacement, and the loss of psychosocial support, among other factors.^[29]^

Post-traumatic stress disorder (PTSD) is another mental health disorder that has been commonly reported among people who have experienced armed conflict, which is a traumatic experience. In a study conducted among displaced persons during the armed conflict in Nepal, it was found that nearly all participants reported trauma, with half reporting symptoms of PTSD.^[30]^ Additionally, 8 in 10 participants in the same study experienced anxiety and depression, which are also common mental health disorders associated with armed conflicts.^[31]^ Other mental health disorders have been reported, particularly among children who are prone to developing a wide range of behavioral problems.^[32]^ A systematic review of armed conflicts indicates a higher prevalence of attention deficit hyperactivity disorder, obsessive-compulsive disorder, generalized anxiety disorder, oppositional defiant disorder, and separation anxiety.^[33]^ Adolescents were also found to engage in high-risk behaviors, such as disobeying parents, dangerous driving, and conduct disorder.^[34]^ Childhood and adolescence are critical periods in a child’s development, and psychological trauma from armed conflict can have a wide range of effects on them, given their susceptibility.^[34]^

## 3. Strategies for safeguarding public health in conflict zones

While matters of political and diplomatic importance dominate discussions in times of conflict, public health issues are often swept under the carpet.^[35,36]^ Thus, the health of people continues to be threatened by infections, physical injuries and psychosocial derangements. However, due to the health risks of armed conflicts, strategies for safeguarding public health in conflict zones require a robust approach.

### 3.1. Humanitarian response in conflict areas

During conflicts, huge pressure exists on affected areas’ social and health infrastructure.^[37]^ Thus, international organizations often spearhead humanitarian efforts to ensure the socioeconomic and health needs of the people in those areas are met. The significance of entities such as the United Nations and the International Committee of the Red Cross is not only felt regarding health. They also collaborate with relevant stakeholders to ensure humanitarian workers’ safe access to conflict areas ^[38]^. In addition, International organizations facilitate training of local response teams to ensure adequate provision of health support to those in need, thus strengthening local health systems.^[38]^ Effective coordination with local authorities helps international organizations to provide effective humanitarian response in times of conflict. This is because the local authorities understand the terrain and geography of the conflict zones, making it easy to facilitate the execution of humanitarian plans. Also, collaboration with local authorities ensures safe security for healthcare workers, aiding the provision of needed medical services in affected areas.^[39]^

### 3.2. Protection of healthcare workers and infrastructure

Protecting healthcare workers is another strategy to safeguard public health in conflict zones. According to international humanitarian laws and the Geneva Convention, the safety of healthcare workers is paramount during conflicts and should be prioritized.^[40]^ One way to achieve that is to create safe zones where health workers can provide services to those in need. In addition, protecting health infrastructure from damage is equally important. Achieving this includes providing security coverage for healthcare centers and facilities.^[41]^ However, in most conflict zones, the health systems are crippled, making it difficult for health workers to provide medical services to wounded military personnel and the civilian population.^[42]^ Thus, there is a need for stringent adherence to the international regulations guiding the provision of medical services in conflict zones and the attachment of strong sanctions to violators of such regulations.

### 3.3. Building preparedness and resilience in conflict-prone regions

Building preparedness and resilience is a major act of strengthening the health system in the long term. This proactive approach involves strengthening the health system and improving its readiness to withstand disasters like wars and conflicts.^[43]^ One of the strategies employed is capacity building, which entails training and re-training healthcare providers and creating community-based health programmes. These acts help to boost the strength of the local health system, increasing its ability to respond to emergencies.^[44]^ Also, providing facilities to cater to people’s health needs in conflict-prone zones is essential for ensuring preparedness to respond to emergencies. Measures should be implemented to provide shelter, food and potable water to displaced people from conflict zones. In addition, ensuring the availability of essential drugs and incorporating mental health services into primary health care helps address people’s immediate health needs. By tailoring the provision of preparedness services to the peculiar needs of people in conflict zones, governments and other stakeholders can effectively provide health responses to victims of wars and civilian populations.

### 3.4. Promoting mental health support and psychosocial interventions

Beyond its physical impact, the conflict also causes significant mental stress on people in conflict zones, a case study being the war between Russia and Ukraine. Thus, promoting mental health support and psychological interventions are important strategies for Promoting public health in these areas. To establish comprehensive mental health support services, collaboration is needed from relevant trained professionals such as psychologists and counselors. These services benefit not only the direct victims of conflicts but also the healthcare professionals working on the battlefield to save lives. Psychosocial interventions for victims in conflict zones include providing clinical support. It also encompasses the provision of community development services and support groups. These interventions are targeted at providing services that improve coping strategies, facilitate positive thinking, and provide social support for people suffering from mental and psychological traumas.

## 4. Conclusion

The surge in armed conflicts globally, as evidenced by the alarming number of state-based conflicts in 2022, poses a profound threat to public and global health. The repercussions of these conflicts, spanning continents and escalating in intensity, are akin to a “pandemic” with far-reaching implications for trauma, infectious diseases, malnutrition, and mental health. The toll on healthcare systems is evident, with deliberate targeting of health facilities exacerbating the challenges. This paper explored the health impacts of armed conflicts, from physical injuries and infectious diseases to the vulnerability of children to malnutrition. The mental health consequences are equally distressing, with trauma, anxiety, depression, and disorders such as PTSD affecting individuals, especially in conflict-affected regions. As the world deals with this “pandemic” of armed conflicts, strategies for safeguarding public health in conflict zones are imperative. Humanitarian response, collaboration with international organizations, and protection of healthcare workers and infrastructure are crucial. Building preparedness and resilience in conflict-prone regions and promoting mental health support and psychosocial interventions form a comprehensive approach. The health and well-being of populations in conflict zones demand attention, and concerted efforts are essential to mitigate the dire consequences of this “pandemic” and pave the way for a healthier, more secure global future.

## Author contributions

**Conceptualization:** Emmanuel Kokori, Gbolahan Olatunji, Nicholas Aderinto.

**Data curation:** Emmanuel Kokori, Nicholas Aderinto.

**Visualization:** Nicholas Aderinto.

**Writing – original draft:** Emmanuel Kokori, Gbolahan Olatunji, Ismaila Ajayi Yusuf, Timilehin Isarinade, Abdulrahmon Moradeyo Akanmu, Doyin Olatunji, Olumide Akinmoju.

**Writing – review & editing:** Emmanuel Kokori, Gbolahan Olatunji, Ismaila Ajayi Yusuf, Timilehin Isarinade, Abdulrahmon Moradeyo Akanmu, Doyin Olatunji, Olumide Akinmoju.
